# Targeting of ribosomal protein S6 to dendritic spines by *in vivo* high frequency stimulation to induce long-term potentiation in the dentate gyrus

**DOI:** 10.1242/bio.013243

**Published:** 2015-10-02

**Authors:** Itsuko Nihonmatsu, Noriaki Ohkawa, Yoshito Saitoh, Kaoru Inokuchi

**Affiliations:** 1 Mitsubishi Kagaku Institute of Life Sciences, MITILS, 11 Minamiooya, Machida, Tokyo 194-8511, Japan; 2 Japan Science and Technology Agency, Core Research for Evolutional Science and Technology, Honcho 4-1-8, Kawaguchi 332-0012, Japan; 3 Department of Biochemistry, Graduate School of Medicine and Pharmaceutical Sciences, University of Toyama, 2630 Sugitani, Toyama 930-0194, Japan

**Keywords:** Ribosomal protein S6, Hippocampus, Long-term potentiation (LTP), Local protein synthesis, Dendritic spine

## Abstract

Late phase long-term potentiation (L-LTP) in the hippocampus is believed to be the cellular basis of long-term memory. Protein synthesis is required for persistent forms of synaptic plasticity, including L-LTP. Neural activity is thought to enhance local protein synthesis in dendrites, and one of the mechanisms required to induce or maintain the long-lasting synaptic plasticity is protein translation in the dendrites. One regulator of translational processes is ribosomal protein S6 (rpS6), a component of the small 40S ribosomal subunit. Although polyribosomes containing rpS6 are observed in dendritic spines, it remains unclear whether L-LTP induction triggers selective targeting of the translational machinery to activated synapses *in vivo*. Therefore, we investigated synaptic targeting of the translational machinery by observing rpS6 immunoreactivity during high frequency stimulation (HFS) for L-LTP induction *in vivo*. Immunoelectron microscopic analysis revealed a selective but transient increase in rpS6 immunoreactivity occurring as early as 15 min after the onset of HFS in dendritic spine heads at synaptic sites receiving HFS. Concurrently, levels of the rpS6 protein rapidly declined in somata of granule cells, as determined using immunofluorescence microscopy. These results suggest that the translational machinery is rapidly targeted to activated spines and that this targeting mechanism may contribute to the establishment of L-LTP.

## Abstract (precis)

**Summary:** Induction of long-term potentiation, which is a cellular model of learning and memory, mediates the selective targeting of the translational machinery on activated dendritic spines *in vivo*.

## INTRODUCTION

New protein synthesis mediated by neural activity is an essential process for long-lasting neuronal plasticity. Late phase long-term potentiation (L-LTP) in the hippocampus is one type of neural plasticity, and it requires new protein synthesis (Frey and Morris, 1997; Nguyen and Kandel, 1997). The synaptic tagging theory explains input-specific functioning at the activated synapses for proteins that are newly synthesized at the soma (Frey and Morris, 1997; Okada et al., 2009). In addition to the *de novo* protein synthesis in the soma, some synaptic proteins are synthesized locally from mRNAs that are present in the dendrites in an activity-dependent manner (Bramham and Wells, 2007).

A population of dendritic RNA is redistributed by neural activity (Matsumoto et al., 2007), and dendritic translation contributes to the establishment of L-LTP (Bradshaw et al., 2003; Miller et al., 2002). The polyribosome, an mRNA with multiple ribosomes attached, is associated with translation, and because polyribosomes are located at dendritic spines (Steward and Levy, 1982), it is believed that protein synthesis is regulated at synapses. To locally synthesize proteins at activated synaptic sites, translational machinery must be targeted to dendritic spines during the expression of plasticity. *In vitro* studies revealed that polyribosomes are selectively increased in spines during LTP in the CA1 region of slices prepared from the developing and mature hippocampus (Bourne et al., 2007; Ostroff et al., 2002). However, it remains unclear whether LTP induction *in vivo* also triggers the selective targeting of the translational machinery to activated sites, and whether the selective targeting is directed by stimulation inducing LTP in hippocampal areas outside the CA1 region.

In the hippocampal dentate gyrus, each granule cell receives two inputs from the entorhinal cortex, one each from the medial and lateral perforant pathways (MPP and LPP, respectively). The MPP and LPP share the lamination domain of the molecular layer (ML), the middle one-third (MML) and outer one-third (OML), respectively. Moreover, each granule cell also receives a portion of the major hilar projection at the inner one-third of ML (IML) (Steward, 1976; Tamamaki, 1999). Although newly synthesized activity-regulated cytoskeleton-associated protein (*Arc*) mRNA induced by neural activation at the MPP–granule cell synapses selectively localizes near the activated synaptic site on the dendrite within 1 h of activation (Steward et al., 1998), a subcellular distribution of the translational machinery has not been reported following synaptic activation of the dentate gyrus *in vivo*.

Ribosomal protein S6 (rpS6) is one of the molecules contained in the 40S ribosome. The rpS6 is found in polyribosome-enriched fractions prepared from cultured cortical neurons (Krichevsky and Kosik, 2001). The mRNAs that contain the 5′ terminal oligopyrimidine tract (5′TOP) sequence in their 5′ untranslated region encode for ribosomal proteins and certain other components of the translational machinery, such as the elongation factors eEF1A and eEF2 (Klann et al., 2004; Tsokas et al., 2007). The rpS6 is also translated from 5′TOP mRNA (Klann et al., 2004; Tsokas et al., 2007). In CA1 pyramidal cells, the translation of dendritic mRNAs that contain 5′TOP sequences is upregulated by L-LTP-inducing stimulation at CA3–CA1 synapses *in vitro*. In addition, translational factors, including rpS6, increase in dendritic regions that receive high frequency stimulation (HFS) to induce translation-dependent LTP (Tsokas et al., 2007). This increase is regulated by the mitogen-activated protein kinase cascade through the mammalian target of rapamycin pathway (Tsokas et al., 2007). HFS of the MPP increases the expression levels of the translation factors, including rpS6, in the dentate gyrus *in vivo* (Panja et al., 2009). These observations strongly suggest that monitoring the rpS6 expression pattern will help clarify the relationship between L-LTP consolidation and the distribution of the local translational machinery.

In this study, we used immunoelectron microscopy to investigate whether L-LTP-inducing stimulation regulates targeting of rpS6 to dendritic spines. Our observations indicate that the translational machinery containing rpS6 is selectively but transiently targeted to the L-LTP-induced spines after HFS *in vivo*. Our results suggest that the translational machinery is rapidly targeted to activated spines, and this targeting mechanism may be one of the most important processes for establishing L-LTP.

## RESULTS

### L-LTP-inducing HFS rapidly and specifically increases F-actin content in the stimulated layers

**Fig. 1.**
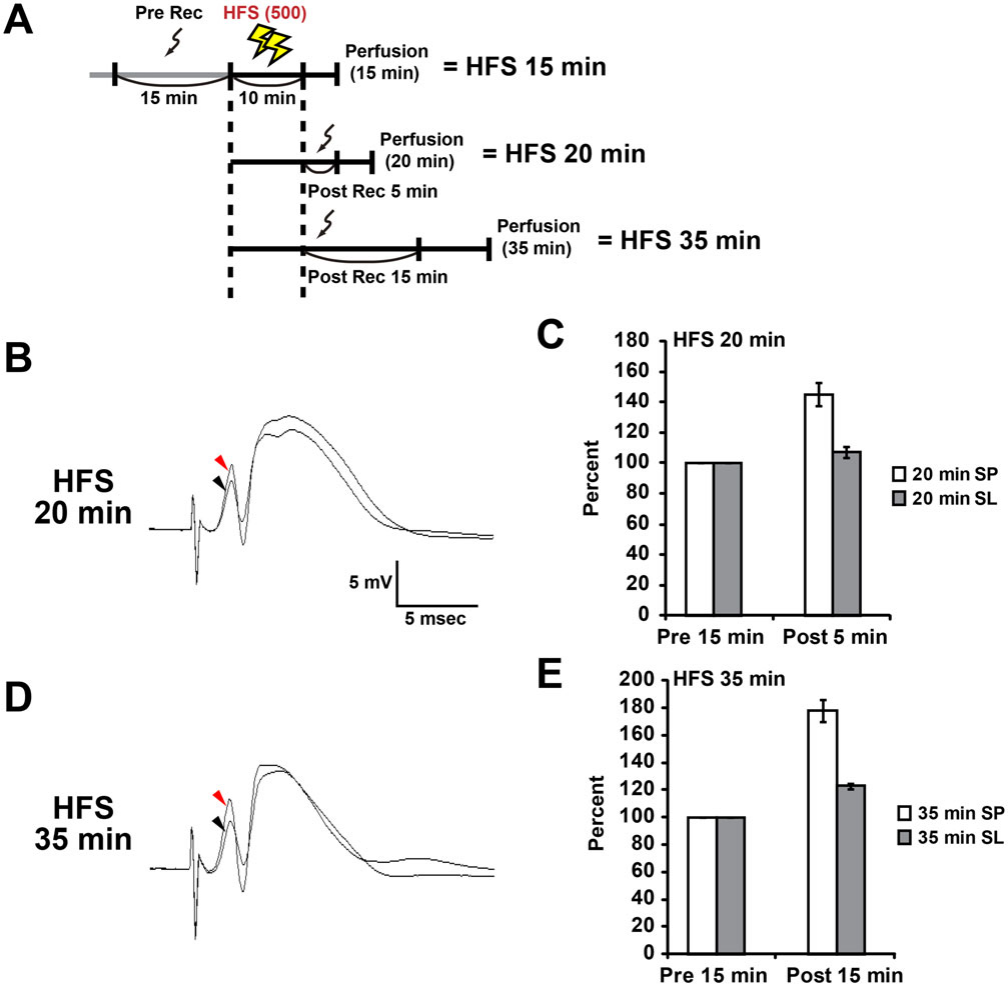
**High frequency stimulation (HFS)-induced L-LTP potentiates spike amplitude and field EPSP slope in the dentate gyrus.** (A) Experimental schedule for this study. Sampling was performed after transcardial perfusion of paraformaldehyde. The perfusion began 15, 20 or 35 min after the start of HFS (10 min). (B-E) Data obtained at 20 min (B,C) and 35 min (D,E) under HFS conditions. (B,D) Typical waveforms pre- and post-HFS are indicated by black and red arrowheads, respectively. (C,E) Relative changes in population spike amplitude (SP) and field EPSP slope (SL) after HFS. Data from each condition were obtained from three animals. Data shown as mean±s.e.m.

**Fig. 2.**
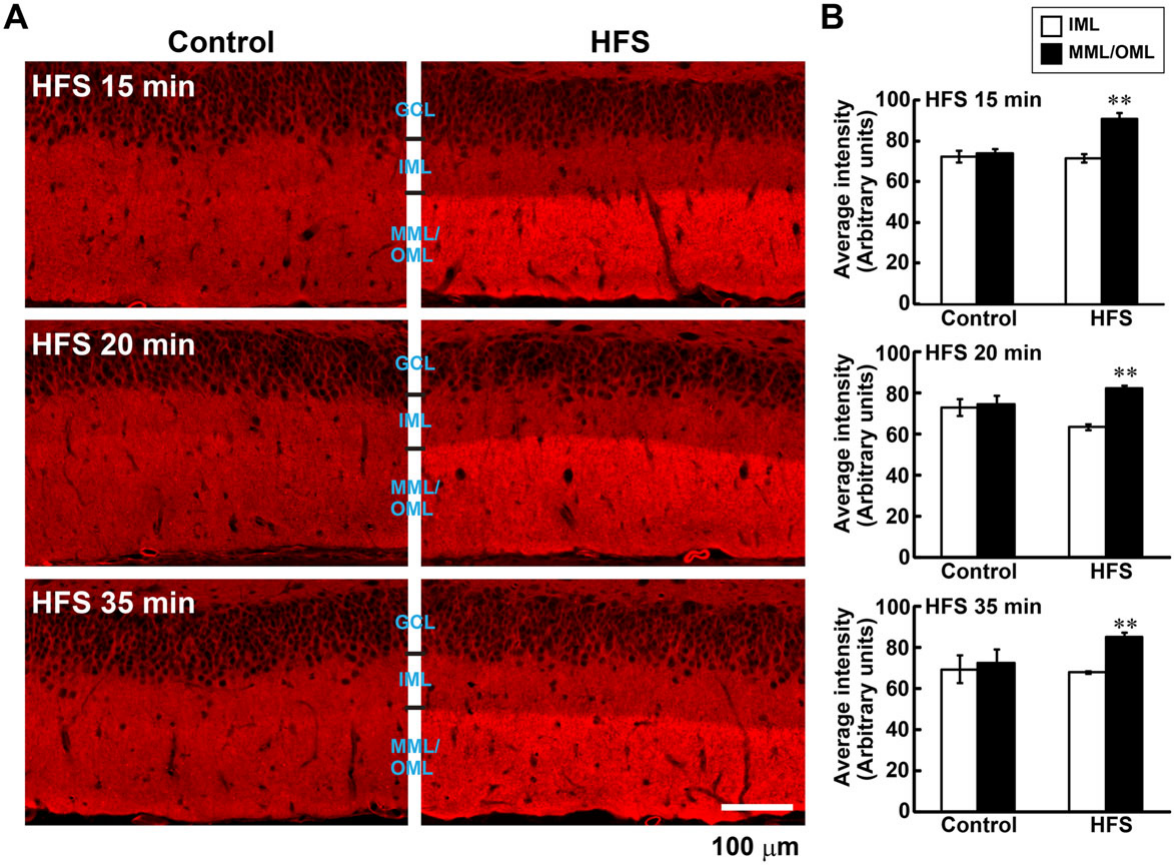
**Expression of F-actin after L-LTP-inducing high frequency stimulation (HFS) in the dentate gyrus.** (A) Micrographs showing phalloidin-rhodamine staining of F-actin in the dentate gyrus. Left panels, control hemisphere; right panels, HFS hemisphere. Scale bar: 100 µm. (B) F-actin staining is selectively increased in laminae that received HFS (middle/outer molecular layers, MML/OML). Graphs show the average intensity of F-actin in the lower blade of the dentate gyrus. Data from each time point were obtained from three animals. Error bars indicate mean±s.e.m. **P<0.01 versus inner molecular layer (IML) using Student's *t*-test.

We previously reported that HFS(500)-induced L-LTP in the dentate gyrus of freely moving rats is associated with the reorganization of the actin cytoskeleton, characterized by a long-lasting increase in F-actin content within the LTP-induced layer (Fukazawa et al., 2003; Ohkawa et al., 2012). The increase in F-actin levels was observed from 45 min to several weeks after the onset of HFS, suggesting that this increase is important in the late phase of LTP (Fukazawa et al., 2003). Here, we first investigated whether the expression of F-actin in the L-LTP-induced dentate gyrus is altered earlier than 45 min after the onset of HFS(500) with three conditions in which sampling were performed at 15, 20, and 35 min after the onset of HFS(500) (HFS 15 min, 20 min, and 35 min in Fig. 1A). A potentiation was observed in the PS amplitude and field EPSP slope 15 and 25 min after the onset of HFS (Fig. 1B-E, from HFS 20 min and 35 min conditions) for animals in the present study. Although we could not record evoked field potentials because of the limited time between the end of HFS and the onset of transcardial perfusion (Fig. 1A), histochemical examinations using phalloidin, a specific probe for F-actin, revealed that the F-actin content rapidly increased in the MML and OML 15 min after the onset of HFS (Fig. 2A). The increase in phalloidin reactivity was observed in the lower blade of the dentate gyrus for both the MML and OML in all animals receiving HFS (Fig. 2A). Therefore, we measured the average intensities of the F-actin signal in the IML, MML, and OML of the lower blade. Quantitative analyses indicated that the F-actin content was significantly elevated in the MML and OML compared with that in the IML at 15 min, 20 min, and 35 min for the hemisphere receiving the HFS (Fig. 2B). However, the level of phalloidin reactivity in the MML and OML was comparable to that in the IML on the control side (Fig. 2B). These data strongly indicated that HFS was indeed delivered to the MML and OML of the lower blade. These results also suggested that the resulting L-LTP induced *in vivo* in those layers was accompanied by a rapid increase in the F-actin content.

### Ribosomal S6 protein transiently decreases in the somata of granule cells after L-LTP-inducing HFS

**Fig. 3.**
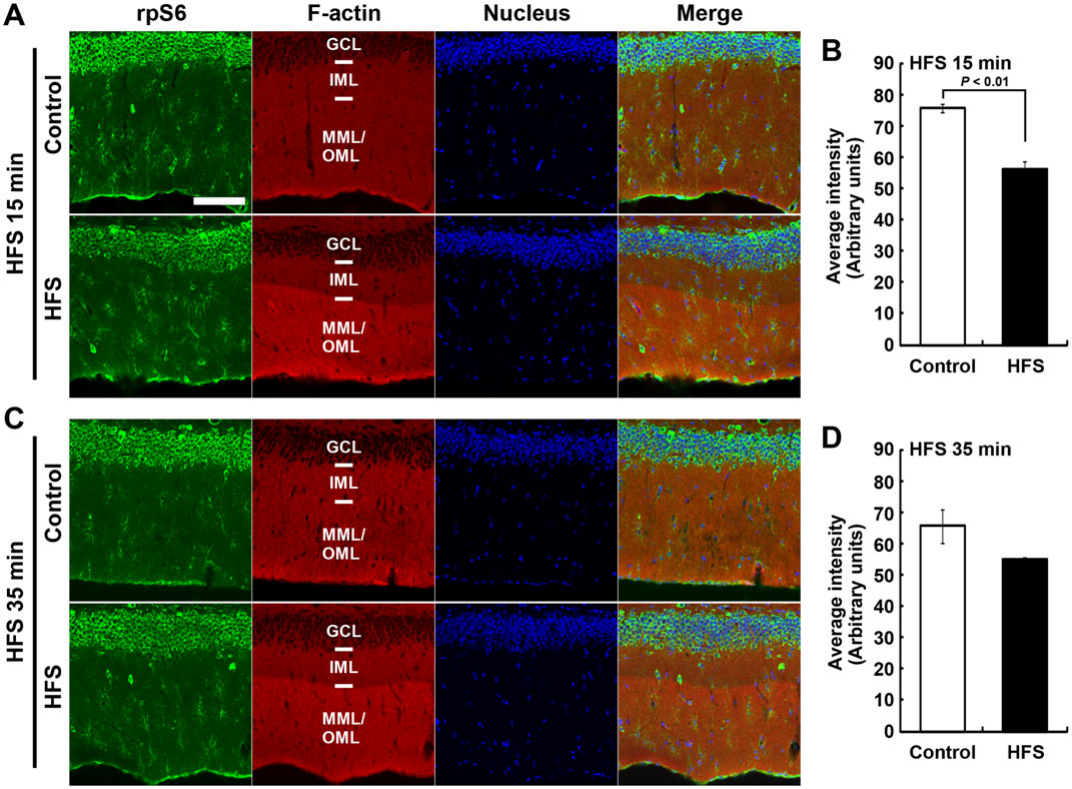
**Ribosomal protein S6 (rpS6) immunofluorescence and F-actin staining distribution after high frequency stimulation (HFS).** (A,C) Micrographs of rpS6 immunostaining, F-actin staining with phalloidin-rhodamine, and DAPI nuclear staining in the dentate gyrus, obtained from 15 min (A) and 35 min (C) under HFS conditions. Scale bar: 100 µm. (B,D) Graphs show average signal intensity of rpS6 in the granule cell layer (GCL). Data obtained from 15 min (B) and 35 min (D) under HFS conditions. Error bars indicate mean±s.e.m. The *P* value from Student's *t*-test is shown in panel B.

To investigate changes in the expression pattern of the translational machinery following LTP induced *in vivo*, we examined the distribution of rpS6 signals using immunofluorescence microscopy following HFS-induced L-LTP in the lower blade of the dentate gyrus. In the control dentate gyrus, strong rpS6 signals were observed in the granule cell layer, where somata of granule cells are localized (Fig. 3A,C). The rpS6 signals were decreased following HFS, and this decrement was significant at 15 min after the onset of HFS (arbitrary units; Control, 75.5±1.4; HFS, 56.1±2.4; *P*<0.01, Student's *t*-test; *n*=3; Fig. 3A,B), but not at 35 min after the onset of HFS (arbitrary units; Control, 65.6±5.3; HFS, 55.0±0.7; *P*>0.11, Student's *t*-test; *n*=3; Fig. 3C,D). The rpS6 signal was diffusely expressed in the ML under the confocal laser-scanning microscope; however, some cells in the ML strongly expressed rpS6 immunoreactivity. Therefore, we next observed rpS6 expression in granule cells using immunoelectron microscopy.

### Ribosomal S6 protein is targeted to dendritic spines in the MML and OML following L-LTP-inducing HFS

**Fig. 4.**
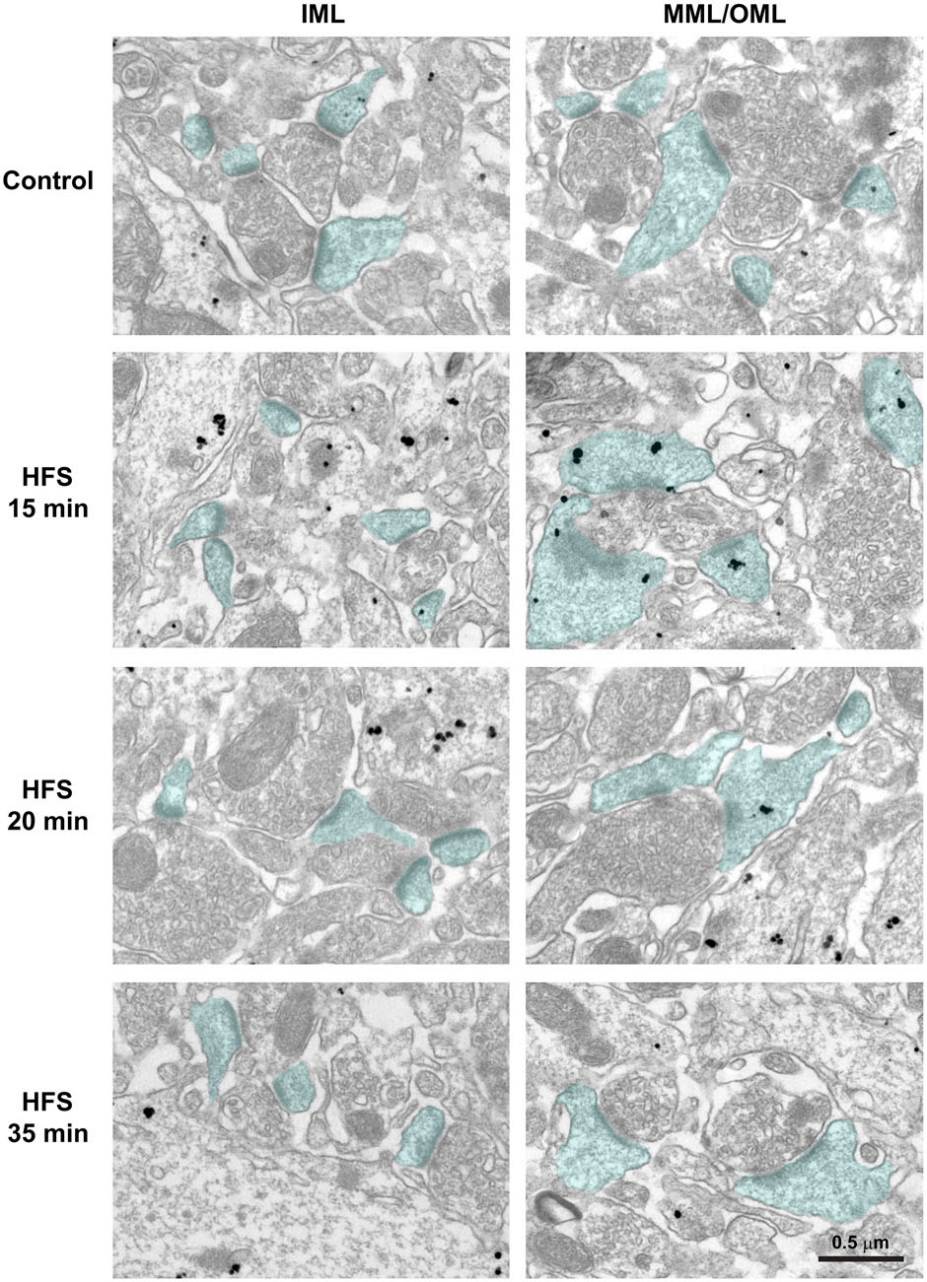
**Immunoelectron microscopic observation of ribosomal protein S6 (rpS6) distribution after high frequency stimulation (HFS).** Immunohistochemistry was performed with hippocampal dentate gyrus sections from the contralateral hemisphere and the ipsilateral hemisphere of HFS 15 min, 20 min, or 35 min condition. Micrographs of inner molecular layer (IML) (left panels) and middle/outer molecular layers (MML/OML) (right panels) are shown. Nanogold particles indicate rpS6 signals. Spines containing postsynaptic densities are blue. Scale bar: 0.5 µm.

The early decrease in the strong rpS6 immunofluorescence signal in somata after the HFS-induced L-LTP suggested HFS-dependent movement of rpS6 from the soma to other subcellular regions. A previous *in vitro* study revealed that the translational machinery, including rpS6, increases in dendritic regions receiving HFS (Tsokas et al., 2007), and polyribosomes are selectively increased in spines during LTP in the hippocampal CA1 region. However, it was unclear whether LTP induction *in vivo* triggers the selective targeting of the translational machinery to activated synaptic sites. We examined the distribution of rpS6 in spines using immunoelectron microscopy following L-LTP induction in the dentate gyrus, because rpS6 is contained in the polyribosome-enriched fractions prepared from cultured cortical neurons (Krichevsky and Kosik, 2001). In the present study, immunoelectron microscopic observation revealed that the number of immunogold particles in spines in the IML showed no change following HFS compared with that in the control (Fig. 4, left panels). By contrast, the number of rpS6 immunoreactive signals in spines clearly increased in both the MML and OML (MML/OML) 15 min after the onset of HFS (HFS 15 min in Fig. 4, right panels).

**Fig. 5.**
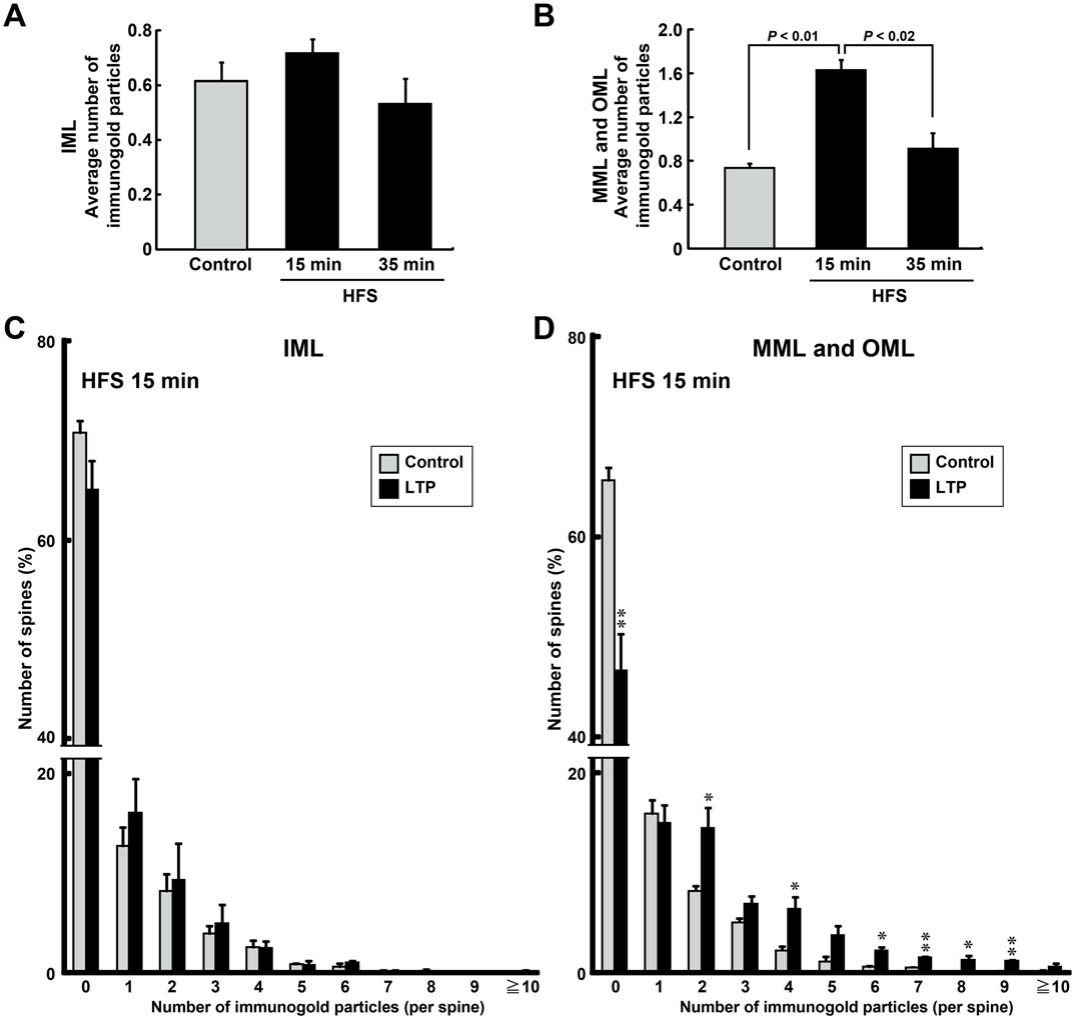
**Ribosomal protein S6 (rpS6) is selectively and transiently distributed to spines after HFS.** (A,B) Graphs of the average number of nanogold particles within each spine of each animal are shown. Data obtained from the inner molecular layer (IML) (A) and middle/outer molecular layers (MML/OML) (B). Error bars indicate mean±s.e.m. *P* values from Scheffé's post hoc test are shown in panel B. (C,D) Histograms indicate percentage of spines that contain each number of nanogold particles. Average data for each condition were obtained from each animal. (A-D) The number of nanogold particles present in each spine was quantified. Control: IML, *n*=292, 374, and 130 spines from *n*=8, 9, and 4 images, respectively; MML and OML, *n*=912, 765, and 597 spines from *n*=25, 24, and 13 images, respectively. HFS 15 min: IML, *n*=273, 79, and 128 spines from *n*=9, 3, and 5 images, respectively; MML and OML, *n*=843, 295, and 170 spines from *n*=24, 9, and 6 images, respectively. HFS 35 min: IML, *n*=152, 82, and 48 spines from *n*=4, 3, and 4 images, respectively; MML and OML, *n*=484, 252, and 69 spines from *n*=13, 10, and 8 images, respectively. *n*=3 animals for each condition. Error bars indicate mean±s.e.m. *P<0.05, **P<0.01 versus control using Student's *t*-tests.

Therefore, we quantified the number of immunogold signals on spines using electron micrographs. In the IML, no differences were detected between the control and LTP-induced (HFS 15 or 35 min) hemispheres for those spines containing signals in the average number of particles on each spine (Control, 0.61±0.07; HFS 15 min, 0.72±0.05; HFS 35 min, 0.53±0.09; *F*_(2,6)_=1.77, *P*>0.249, ANOVA; *n*=3 animals for each condition) (Figs 4, 5A). In the MML/OML, no significant differences were found in the average number of particles on each spine between the control and LTP-induced hemispheres 35 min after starting the LTP-inducing HFS (Control, 0.73±0.04; HFS 35 min, 0.96±0.15; *n*=3 animals) (Figs 4, 5B). By contrast, the average number of immunogold particles was significantly increased in the MML/OML 15 min after starting HFS (1.63±0.09; *n*=3 animals) compared with that in the control hemisphere or that 35 min after starting HFS (*P*<0.01, ANOVA; HFS 15 min vs Control, *P*<0.01; HFS 15 min vs HFS 35 min, *P*<0.02; Scheffé's post hoc test) (Figs 4, 5B). This increase in the average number of particles on each spine in HFS 15 min was due mainly to a significant decrease in the percentage of spines without rpS6 signals (MML/OML: Control, 1874 spines, 65.67%±1.28; HFS 15 min, 1308 spines, 46.67%±3.67; *P*<0.01, Student's *t*-test; *n*=3 animals) (Fig. 5D) and also due to an increase in the percentage of the spines with multi-particles (Fig. 5D). By contrast, no difference was detected in the percentage distribution of spines in the IML that contained immunogold particles between the control hemisphere and the hemisphere receiving HFS (Control, 796 spines; HFS 15 min, 480 spines; *n*=3 animals) (Fig. 5C). These results suggested that the translational machinery was rapidly and transiently translocated from the soma to the activated spines and/or synthesized in the activated synapses.

## DISCUSSION

rpS6 is a major marker for ribosome. Indeed, rpS6 was identified as one of eukaryotic 40S ribosomal subunits (Rabl et al., 2011). The rpS6 was specifically distributed in the fractions with ribosome and polyribosome, but not in fraction of mRNPs (mRNA-protein complexes), of neuronal cells from rat forebrain (Krichevsky and Kosik, 2001). In addition, it was mentioned that “rpS6 was very low in amount in the soluble fraction of the forebrain” (Asaki et al., 2003). These evidences strongly suggest that majority of rpS6s were contained in ribosomal complexes, but not distributed freely in cytoplasm. Other translational proteins, eIF4E and ribosomal S3 protein, were also detected in dendritic spines by immunoelectron microscopy. Presence of polyribosome was actually revealed in the dendritic spines, and about 30% of the spines contained the polyribosome in basal state (Steward and Levy, 1982). This percentage is quite comparable with our present data of spine with rpS6 signal in control inner molecular layer (IML) and middle/outer molecular layer (MML/OML) where dendrites of granule cells locate (Fig. 5C,D, control). Both studies, Steward et al. and our study, analyzed granule cells of dentate gyrus in rats. Taken together, these evidences strongly suggest that rpS6 signals reflect the presence of ribosome and polyribosome in dendritic spines of the granule cells in dentate gyrus.

In this study, we investigated the distribution of the translational machinery that includes rpS6 after *in vivo* L-LTP induction. Delivery of HFS induced an increase in levels of F-actin at the induced lamina in the lower blade of the dentate gyrus. This increase in F-actin levels was maintained beginning 15 min after the onset of HFS (Fig. 1). Concurrently, rpS6 immunoreactive signals decreased in the soma and selectively increased in dendritic spines in the lamina of the lower blade of the dentate gyrus 15 min after HFS onset (Figs 3, 4, 5). However, unlike the sustained increase in F-actin levels, the observed intensity of rpS6 immunostaining in spines rapidly returned to initial levels 35 min after the onset of HFS (Figs 4, 5). These results indicate that the translational machinery is selectively, immediately, and transiently targeted to spines at sites of HFS-induced LTP *in vivo*.

Alpha calcium/calmodulin-dependent protein kinase II (αCaMKII) is an important factor required for the establishment of hippocampal LTP and hippocampus-dependent learning (Giese et al., 1998; Silva et al., 1992a,b), and inhibition of the dendritic translocation of *αCaMKII* mRNA suppresses hippocampal L-LTP (Miller et al., 2002). HFS in the dentate gyrus of freely moving rats triggers rapid and transient delivery of the pre-existing *αCaMKII* mRNA into synaptodendrosomes (Havik et al., 2003). Depolarization of the synaptosomal membrane enhances the synthesis of αCaMKII protein (Bagni et al., 2000). Both of these events are observed until 45 min after each stimulation. These results suggest that local protein synthesis in dendritic spines is triggered at a relatively early phase during the establishment of L-LTP.

It remains unclear which mechanisms function for the rapid movement of translation machinery after HFS. Depolarization of the synaptosomal membrane increases the association between *αCaMKII* mRNA and polysomes (Bagni et al., 2000). The motility of *CaMKII* mRNA is rapidly decreased by neural activation, and the mRNA then transiently increases in dendritic spines within approximately 10 min (Kao et al., 2010). Therefore, the expression pattern of rpS6 after HFS may depend not only on the targeting of the mRNA to dendritic spines but also on a fluctuation in the association affinity between the locally translatable mRNA and the translational machinery.

Ribosomal assembly reportedly requires rpS6 (Volarevic et al., 2000). The expression of several translation factors is increased during the immediate early phase of several types of LTP induced by HFS (Panja et al., 2009; Tsokas et al., 2007). This evidence combined with that in our present report, which shows a translocation of translation machinery immediately after HFS, suggests that the translocated translation machinery may initiate *de novo* production of translation machinery at the LTP-induced sites *in vivo*. This *de novo* production may contribute not only to local protein synthesis but also to a cell-wide process that establishes L-LTP by increasing the total translational capacity. This cell-wide process is consistent with the synaptic tagging theory, which explains the input-specific functioning of newly synthesized proteins at activated synapses (Frey and Morris, 1997; Okada and Inokuchi, 2015; Okada et al., 2009). However, it was suggested that the increase in the expression of rpS6 and other translational factors after HFS is not required for LTP maintenance 4 h after the induction (Panja et al., 2009). Thus, the rpS6-mediated local translation may be required only during LTP consolidation, whereas an overall cell-wide increase in translational capacity may function in the later stages of LTP maintenance.

## MATERIALS AND METHODS

### Animals

All procedures involving the use of animals complied with the guidelines recommended by the National Institutes of Health and were approved by the Animal Care and Use Committees of Mitsubishi Kagaku Institute of Life Sciences and University of Toyama. Male Wistar ST rats (Japan SLC Inc., Shizuoka, Japan) approximately 20 weeks of age were used for LTP experiments.

### Dentate gyrus LTP induction in unanesthetized freely moving animals

The surgical procedures used in this study were described previously (Fukazawa et al., 2003; Kato et al., 1998, 1997; Ohkawa et al., 2012). The electrode stimulating the PP fibers was positioned 8.0 mm posterior, 4.5 mm lateral, and 5.0 mm inferior to bregma. A recording electrode was implanted ipsilaterally 4.0 mm posterior, 2.5 mm lateral, and 3.8 mm inferior to bregma.

LTP experiments on freely moving animals were performed as described previously (Fukazawa et al., 2003; Matsuo et al., 2000; Ohkawa et al., 2012). LTP was induced using tetanic stimuli with biphasic square waveforms at a pulse width of 200 µs. The maximal population spike (PS) amplitude was determined, and the intensity of the stimulus current was set to elicit 60% of the maximal PS amplitude. This intensity was used for baseline recording and 500 pulses of HFS [HFS(500)] experiments. The animal was transferred to the recording chamber, and the baseline response was monitored by delivering test pulses (0.05 Hz) for 15 min (Pre Rec, Fig. 1A). After baseline monitoring, LTP was induced using strong high frequency tetanic stimulation, HFS(500), consisting of 10 trains with 1 min intertrain intervals (total, 10 min). Each train consisted of five bursts of 10 pulses at 400 Hz, delivered at 1 s interburst intervals. Synaptic transmission was monitored for 5 or 15 min after delivery of HFS(500) in case of HFS 20 min or HFS 35 min, except for the sampling at 15 min after HFS onset, HFS 15 min (Fig. 1A).

### Histochemistry

The rats were deeply anesthetized with an overdose of pentobarbital solution and perfused transcardially with phosphate-buffered saline (PBS), pH 7.4, followed by 4% paraformaldehyde (PFA) in PBS. Their brains were removed and postfixed by immersion in 4% PFA in PBS for 2 h at 4°C. Each brain was equilibrated in 25% sucrose in PBS and then frozen in powdered dry ice. Coronal sections were cut on a cryostat at a thickness of 14 µm. After being washed with PBS, the sections were treated with PBS supplemented with 0.5% Triton X-100 at room temperature (RT) for 20 min, followed by two 10 min washes with PBS. The sections were then treated with blocking buffer (5% goat serum in PBS supplemented with 0.1% Triton X-100) at RT for 1 h. Reactions with primary antibodies were performed in blocking buffer containing rabbit anti-rpS6 polyclonal antibody (1:100; Cell Signaling Technology, Beverly, MA, USA) at 4°C overnight. After three 10 min washes with PBS, the sections were incubated with Alexa Fluor 488-conjugated anti-rabbit secondary antibody (1:100; Invitrogen, Carlsbad, CA, USA) at RT for 3 h. Sections were incubated in phalloidin-TRITC (0.1 ng/ml; Sigma, St. Louis, MO, USA) for 2 h at RT or overnight at 4°C. For nuclear staining, sections were treated with 4′,6-diamidino-2-phenylindole (DAPI) (1 µg/ml) for 20 min at RT. Mounting of sections on slide glasses was performed with ProLong Gold antifade reagent (Molecular Probes, Eugene, OR, USA). The fluorescent signals were examined with a laser-scanning confocal microscope (LSM700, Carl Zeiss, Jena, Germany). Images shown in Figs 2 and 3 were captured with a light microscope (AX-80T, Olympus, Tokyo, Japan) and a laser-scanning confocal microscope, respectively.

### Immunoelectron microscopy

At selected times after delivery of HFS (Fig. 1A), the rats were deeply anesthetized with ether and perfused transcardially with PBS, followed by 4% PFA and 0.05% glutaraldehyde in PBS. Their brains were removed and slabs were postfixed by immersion in 4% PFA in PBS overnight at 4°C. Each brain slab was equilibrated in 25% sucrose solution in PBS, frozen in O.C.T. compound (Miles, Elkhart, IN, USA), and sliced into 10 µm thick sections using a cryostat. The sections were mounted on silane-coated glass slides. Immunoelectron microscopy was conducted as described previously using a modified pre-embedding 1 nm gold particle (nanogold)-silver enhancement method (Mizoguchi et al., 1994). Briefly, the sections obtained as described above were blocked with 20% Block Ace (Sumitomo Dainippon Pharma Co., Osaka, Japan) in PBS containing 0.02% saponin for 10 min, incubated with rabbit anti-rpS6 polyclonal antibody in PBS containing 5% Block Ace and 0.005% saponin for 48 h at 4°C, and then washed three times for 20 min with 5% Block Ace in PBS containing 0.005% saponin. After being washed, the sections were incubated with a goat anti-rabbit IgG Fab fragment labeled with nanogold (Nanoprobes, New York, USA) at a dilution of 1:50 for 24 h at 4°C, and then fixed in 1% glutaraldehyde for 10 min. After washing in 50 µM HEPES buffer (pH 5.8) for 1 h, tissue-bound gold particles were enhanced by incubation with a silver developer (HQ Silver, Nanoprobes) at 20°C for 12 min in the dark. The sections were then postfixed with 0.5% osmium tetroxide in PB for 1 h, dehydrated by passing through a graded series of ethanol and propylene oxide, and embedded in Epon 812. Ultra-thin sections were cut, stained with uranyl acetate and lead citrate, and observed with an electron microscope (JEM-1230, JEOL, Tokyo, Japan).

### Data analysis

Electron micrographs consisted of linear and consecutive images captured from the inner to outer ML of the lower blade of the dentate gyrus. Magnification was 5000×. Using these micrographs, the number of enhanced immunogold particle signals was counted in all spines containing a postsynaptic density and contacting synaptic vesicle-containing structures. The quantitative measurements of F-actin levels in Fig. 2 and rpS6 levels in Fig. 3 were determined using the average signal intensity obtained with Metamorph software (Molecular Devices, Downingtown, PA, USA). All statistical analyses were performed using StatView software (Abacus Concepts, Berkeley, CA, USA). Comparisons between two groups of data were analyzed using Student's *t*-tests. Multiple-group comparisons were assessed using a one-way analysis of variance (ANOVA), followed by Scheffé's post hoc test when significant main effects were detected. Quantitative data are presented as the mean±standard error of the mean (s.e.m.).
